# Favorable prognosis in colorectal cancer patients with co-expression of c-MYC and ß-catenin

**DOI:** 10.1186/s12885-016-2770-7

**Published:** 2016-09-13

**Authors:** Kyu Sang Lee, Yoonjin Kwak, Kyung Han Nam, Duck-Woo Kim, Sung-Bum Kang, Gheeyoung Choe, Woo Ho Kim, Hye Seung Lee

**Affiliations:** 1 Department of Pathology, Seoul National University Bundang Hospital, 173-82 Gumi-ro, Bundang-gu, Seongnam-si, Gyeonggi-do 463-707 Republic of Korea; 2 Department of Pathology, Seoul National University College of Medicine, 103 Daehak-ro (Yongon-dong), Jongno-gu, Seoul 110-799 Republic of Korea; 3 Department of Pathology, Haeundae Paik Hospital, Inje University College of Medicine, 875, Haeun-daero, Haeundae-gu, Busan, 612-896 Republic of Korea; 4 Department of Surgery, Seoul National University Bundang Hospital, 173-82 Gumi-ro, Bundang-gu, Seongnam-si, Gyeonggi-do 463-707 Republic of Korea

**Keywords:** Colorectal cancer, c-MYC, ß-catenin, Immunohistochemistry, mRNA in situ hybridization, Prognosis

## Abstract

**Background:**

The purpose of our research was to determine the prognostic impact and clinicopathological feature of c-MYC and β-catenin overexpression in colorectal cancer (CRC) patients.

**Methods:**

Using immunohistochemistry (IHC), we measured the c-MYC and β-catenin expression in 367 consecutive CRC patients retrospectively (cohort 1). Also, c-MYC expression was measured by mRNA in situ hybridization. Moreover, to analyze regional heterogeneity, three sites of CRC including the primary, distant and lymph node metastasis were evaluated in 176 advanced CRC patients (cohort 2).

**Results:**

In cohort 1, c-MYC protein and mRNA overexpression and ß-catenin nuclear expression were found in 201 (54.8 %), 241 (65.7 %) and 221 (60.2 %) of 367 patients, respectively, each of which was associated with improved prognosis (*P* = 0.011, *P* = 0.012 and *P* = 0.033, respectively). Moreover, co-expression of c-MYC and ß-catenin was significantly correlated with longer survival by univariate (*P* = 0.012) and multivariate (*P* = 0.048) studies. Overexpression of c-MYC protein was associated with mRNA overexpression (ρ, 0.479; *P* < 0.001) and nuclear ß-catenin expression (ρ, 0.282; *P* < 0.001). Expression of c-MYC and ß-catenin was heterogeneous depending on location in advanced CRC patients (cohort 2). Nevertheless, both c-MYC and ß-catenin expression in primary cancer were significantly correlated with improved survival in univariate (*P* = 0.001) and multivariate (*P* = 0.002) analyses. c-MYC and ß-catenin expression of lymph node or distant metastatic tumor was not significantly correlated with patients’ prognosis (*P* > 0.05).

**Conclusions:**

Co-expression of c-MYC and ß-catenin was independently correlated with favorable prognosis in CRC patient. We concluded that the expression of c-MYC and ß-catenin might be useful predicting indicator of CRC patient’s prognosis.

**Electronic supplementary material:**

The online version of this article (doi:10.1186/s12885-016-2770-7) contains supplementary material, which is available to authorized users.

## Background

The c-MYC protein encode by *c-MYC* gene, acts as transcription factor for variable cellular function including proliferation, differentiation, metabolism, survival, and apoptosis [1, 2]. The *c-MYC* gene can promote tumorigenesis in various malignant tumors [3, 4] and mediate the critical role in the colorectal cancer (CRC) progression [5, 6]. Deregulation of *c-MYC* is a consequence of mutations in *APC,* a central hub in early colorectal carcinogenesis [7].

*c-MYC* gene amplification, translocation, and alteration of regulatory molecules are major causes of c-MYC protein overexpression [8, 9]. Previously, other group indicated that *c-MYC* amplification and overexpression was showed in approximately 10 and 70 % in CRC, respectively [10]. These studies have deduced that overexpression of c-MYC is controlled by mechanisms other than gene amplification [10]. In recent years, it has been evident that the mechanism of c-MYC overexpression is not restricted to genetic alterations, such as amplification or translocation, but can also occur as a consequence of abnormalities in regulatory molecules [11]; in CRC, ß-catenin is one such regulatory molecule. It is now well established that *APC* gene mutation, a key driver of adenoma-carcinoma transition, often leads to altered ß-catenin regulation via the well-studied Wnt signaling pathway [12–14]. Regulation of this pathway occurred while changing in nuclear ß-catenin protein levels. A destruction complex maintains a low cytoplasmic concentration of ß-catenin when the Wnt signaling pathway is inactivated. On the contrary, the destruction complex degrades and ß-catenin increases in the cytoplasm, leading to its migration to the nucleus, where it work like a transcriptional factor for *c-MYC* and *cyclin D1* [15, 16]. Recent studies reported CRCs with marked WNT and c-MYC signaling activation as a distinct molecular subtype by gene expression-based CRC classifications, which was associated with relatively better prognosis [17, 18]. It suggests that CRCs with activated c-MYC via Wnt signaling pathway have distinct clinicopathologic characteristics, but it has not been confirmed.

Nevertheless, there were a few researches that reported clinicopathological impact of c-MYC and ß-catenin status in CRC. Their prognostic value for CRC patients remains debatable. A recent study reported that c-MYC protein overexpression obtained by immunohistochemistry (IHC) was significantly correlated with better survival of CRC patients [19]. In contrast, other researchers conducted a meta-analysis showing that the accumulation of nuclear ß-catenin could be a biomarker for advanced stage and worse survival of CRC [20]. However, the correlation between immunohistochemical nuclear ß-catenin expression and patient prognosis is quite controversial. Consequently, it is necessary to further evaluate c-MYC and ß-catenin expression to reach a conclusion about their prognostic value.

Recently, the systemic chemotherapy in CRC has made a remarkable development, and targeted therapy has been used to increase survival in advanced CRC patients [21]. However, targeted therapy has no effect in some CRC patients, despite presenting positivity for target-therapy specific molecular examination [22]. Several researchers have demonstrated that CRC shows a regional heterogeneity in *KRAS*, *EGFR*, and *BRAF* mutation, thus tumor heterogeneity may explain this discrepancy between molecular alteration and responses of targeted therapy [23–25]. Therefore, molecular alterations between the metastatic and primary lesions need to be discovered to enhance the treatment effect of metastatic CRCs.

The aim of our research was to evaluate the clinical implication of c-MYC and ß-catenin in CRC and evaluate their heterogeneity in primary and distant metastatic tumors. We also analyzed the association between c-MYC and ß-catenin status.

## Methods

### Collection of samples

A total of 543 CRC cases of this study had been collected in our previous study [26]. To investigate the clinicopathological significance of c-MYC and ß-catenin expression, we collected 367 consecutive CRC patients who underwent surgery between 2005 and 2006 at Seoul National University Bundang Hospital (cohort 1). Additionally, to evaluate the locational heterogeneity of c-MYC and ß-catenin expression, we collected synchronous or metachronous metastatic 176 CRC patients with who had received surgery between 2003 and 2004, as well as between 2007 and 2009 excluding any patient already enrolled in cohort 1 (cohort 2). Pathologists K.S.L and H.S.L reviewed all the cases. Cancer stage was determined from the American Joint Committee on Cancer (AJCC) 7th edition. Clinical and pathologic information was acquired from hospital medical records including patient’ outcome and survival.

### Tissue array method

Tissue microarray (TMA) was constructed with representative lesions of the donor formalin-fixed paraffin-embedded (FFPE) CRC tissues as previously described [27].

### Immunohistochemistry

**Fig. 1:**
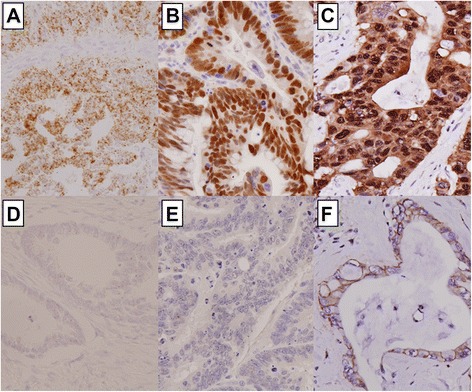
Representative figures of c-MYC status detected by in situ hybridization (**a** and **d**) and immunohistochemistry (IHC; **b** and **e**), and of ß-catenin expression by IHC (**c** and **f**), in colorectal cancer patients. **a** Score 4 mRNA (40×); **b** c-MYC overexpression (40×); **c** Nuclear ß-catenin expression (40×); **d** Score 0 mRNA (40×); **e** No c-MYC expression (40×); **f** Membranous ß-catenin expression (40×)

c-MYC IHC analysis was performed using an antibody against c-MYC (clone Y69, catalog ab32072, Abcam, Burlingame, CA, USA*)*. ß-catenin IHC used a commercially available antibody against ß-catenin (clone CAT-5H10, Invitrogen, Camarillo, CA, USA). The staining process was performed using an automated immunostainer (BenchMark XT, Ventana Medical Systems), according to the manufacturer’s recommendations. Normal colonic mucosa cells were considered as internal negative controls. Normal mucosa was negative for c-MYC nuclear immunostaining. ß-catenin was negative in inflammatory cells, but expressed in colonic epithelium in three patterns: membrane, cytoplasm, and nucleus. We only found ß-catenin nuclear expression in malignant cells. For statistical analysis, c-MYC and ß-catenin immunostaining were regarded as positive when they were expressed in more than 10 % of neoplastic nucleus in any intensity (Fig. 1) [19, 28]. Negative controls were obtained omitting the primary antibody for each immunostaining.

### mRNA in situ hybridization

For the detection of *c-MYC* mRNA transcripts, the RNAscope 2.0 HD detection kit (Advanced Cell Diagnostics, Hayward, CA, USA) was used according to the manufacturer’s protocols. The experimental data was interpreted according to the manual in the RNAscope FFPE assay kit: no staining or less than 1 dot/cell at 40× objective view (score of 0); staining in 1–3 dots/cell visible at 20–40× objective view (score of 1); staining in 4–10 dots/cell with no or very few dot clusters visible at 20–40× objective view (score of 2); staining in >10 dots/cell with less than 10 % of positive cells having dot clusters visible at 20× objective view (score of 3); staining in >10 dots/cell with more than 10 % of positive cells having dot clusters visible at 20× objective view (score of 4). A score of 2–4 indicates *c-MYC* mRNA overexpression (Fig. 1). *UBC* (ubiquitin C) and *dapB* (a bacterial gene) were used for positive and negative controls. Tissues were regarded as appropriate when the *UBC* mRNA signals were visible without difficulty at 10× magnification and the *dapB* signal was not visible.

### Microsatellite instability

Microsatellite instability (MSI) examination using fragmentation assay of ABI-3130xl with five microsatellite markers (BAT-26, BAT-25, D5S346, D17S250, and D2S123) were analyzed according to the instruction demonstrated previously [29]. MSI examination was evaluated in available 519 cases.

### Statistical analyses

All statistical analysis was performed with the SPSS version 21 (IBM, Armonk, NY, USA) software. The Chi-square test or Fisher’s exact test was used for evaluating the correlation between clinicopathological characteristics and c-MYC and ß-catenin expression. The Pearson correlation coefficient was used for analyzing comparison of detection methods. The Kaplan-Meier method with the log-rank test and multivariate regression were performed to assess survival difference. The survival results were determined with hazard ratio (HR) and its 95 % confidence interval (CI). *P* < 0.05 was considered statistically significant.

## Results

### Clinicopathological impacts of c-MYC and ß-catenin expression in consecutive CRC patients

In 367 patients (cohort 1), a *c-MYC* mRNA in situ hybridization score of 0 was observed in 34 (9.3 %), a score of 1 in 92 (25.1 %), a score of 2 in 123 (33.5 %), a score of 3 in 93 (25.3 %), and a score of 4 in 25 (6.8 %). Consequentially, overexpression of *c-MYC* mRNA (a score of 2–4) was observed in 241 patients (65.7 %). c-MYC protein overexpression was observed in 201 (54.8 %), and ß-catenin nuclear overexpression was observed in 221 (60.2 %) patients.

Table 1 demonstrates the correlations between c-MYC and ß-catenin overexpression and clinicopathological parameters. c-MYC protein overexpression was associated with non-aggressive characteristics, including early pT stage, low-grade differentiation, absence of perineural invasion, and smaller tumor size (*P* < 0.001, *P* = 0.007, *P* = 0.025 and *P* < 0.001, respectively). In addition, c-MYC protein overexpression was associated with a tumor location in the recto-sigmoid colon. Increased levels of the *c-MYC* mRNA transcript were associated with microsatellite stable CRC (*P* = 0.019), located in the sigmoid colon and rectum, and with less aggressive features, similarly to c-MYC protein overexpression. Likewise, ß-catenin nuclear expression was frequently detected in tumors of the recto-sigmoid colon, of low-grade differentiation (*P* = 0.006), of small size (*P* = 0.007) and microsatellite stable CRC (*P* < 0.001).

**Table 1:** The association between clinicopathological parameters and expression of c-MYC and ß-catenin in 367 CRC patients (cohort1)

	Total	c-Myc IHC		*P*-Value	*c-Myc* RNA ISH (score)		*P*-Value	ß-catenin IHC (%)		*P*-Value
		Negative	Positive		Negative	Positive		Negative	Positive	
Age										
mean	64.2	64.6	63.9	0.537	63.4	64.7	0.334	64.1	63.7	0.257
Sex										
male	205	89 (43.4 %)	116 (56.6 %)	0.431	72 (35.1 %)	133 (64.9 %)	0.720	82 (40.0 %)	123 (60.0 %)	0.924
female	162	77 (47.5 %)	85 (52.5 %)		54 (33.3 %)	108 (66.7 %)		64 (39.5 %)	98 (60.5 %)	
Location										
cecum	13	12 (92.3 %)	1 (7.7 %)	0.002	12 (92.3 %)	1 (7.7 %)	0.039	11 (84.6 %)	2 (15.4 %)	0.103
ascending colon	55	39 (70.9 %)	16 (29.1 %)		37 (67.3 %)	18 (32.7 %)		36 (65.5 %)	19 (34.5 %)	
hepatic flexure	22	14 (63.6 %)	8 (36.4 %)		17 (77.3 %)	5 (22.7 %)		14 (63.6 %)	8 (36.4 %)	
transverse colon	16	10 (62.5 %)	6 (37.5 %)		11 (68.8 %)	5 (31.3 %)		11 (68.8 %)	5 (31.3 %)	
splenic flexure	6	5 (83.3 %)	1 (16.7 %)		4 (66.7 %)	2 (33.3 %)		4 (66.7 %)	2 (33.3 %)	
descending colon	18	15 (83.3 %)	3 (16.7 %)		15 (83.3 %)	3 (16.7 %)		13 (72.2 %)	5 (27.8 %)	
sigmoid colon	114	53 (46.5 %)	61 (53.5 %)		64 (56.1 %)	50 (43.9 %)		62 (54.4 %)	52 (45.6 %)	
rectum	123	71 (57.7 %)	52 (42.3 %)		89 (72.4 %)	34 (27.6 %)		61 (49.6 %)	62 (50.4 %)	
pT stage										
0–2	58	14 (24.1 %)	44 (75.9 %)	<0.001	14 (24.1 %)	44 (75.9 %)	0.075	17 (29.3 %)	41 (70.7 %)	0.076
3–4	309	152 (49.2 %)	157 (50.8 %)		112 (36.2 %)	197 (63.8 %)		129 (41.7 %)	180 (58.3 %)	
Differentiation										
LG	331	142 (42.9 %)	189 (57.1 %)	0.007	101 (30.5 %)	230 (69.5 %)	<0.001	124 (37.5 %)	207 (62.5 %)	0.006
HG	36	24 (66.7 %)	12 (33.3 %)		25 (69.4 %)	11 (30.6 %)		22 (61.1 %)	14 (38.9 %)	
LN metastasis										
absent	168	67 (39.9 %)	101 (60.1 %)	0.058	58 (34.5 %)	110 (65.5 %)	0.943	63 (37.5 %)	105 (62.5 %)	0.412
present	199	99 (49.7 %)	100 (50.3 %)		68 (34.2 %)	131 (65.8 %)		83 (41.7 %)	116 (58.3 %)	
Lymphatic invasion										
absent	158	63 (39.9 %)	95 (60.1 %)	0.073	51 (32.3 %)	107 (67.7 %)	0.471	61 (38.6 %)	97 (61.4 %)	0.689
present	209	103 (49.3 %)	106 (50.7 %)		75 (35.9 %)	134 (64.1 %)		85 (40.7 %)	124 (59.3 %)	
Perineural invasion										
absent	154	49 (31.8 %)	105 (68.2 %)	0.025	78 (30.7 %)	176 (69.3 %)	0.028	99 (39.0 %)	155 (61.0 %)	0.636
present	113	61 (54.0 %)	52 (46.0 %)		48 (42.5 %)	65 (57.5 %)		47 (41.6 %)	66 (58.4 %)	
Venous invasion										
absent	296	133 (44.9 %)	163 (55.1 %)	0.814	101 (34.1 %)	195 (65.9 %)	0.862	121 (40.9 %)	175 (59.1 %)	0.381
present	71	33 (46.5 %)	38 (53.5 %)		25 (35.2 %)	46 (64.8 %)		25 (35.2 %)	46 (64.8 %)	
Tumor border										
expanding	60	25 (41.7 %)	35 (58.3 %)	0.544	27 (45.0 %)	33 (55.0 %)	0.057	24 (40.0 %)	36 (60.0 %)	0.970
infiltrative	307	141 (45.9)	166 (54.1 %)		99 (32.2 %)	208 (67.8 %)		122 (39.7 %)	185 (60.3 %)	
Size (cm)										
mean	5.3	5.8	4.8	<0.001	6.0	4.9	<0.001	5.6	5.0	0.007
Distant metastasis										
absent	299	131 (43.8 %)	168 (56.2 %)	0.252	97 (32.4 %)	202 (67.6 %)	0.110	115 (38.5 %)	184 (61.5 %)	0.278
present	68	35 (51.5 %)	33 (48.5 %)		29 (42.6 %)	39 (57.4 %)		31 (45.6 %)	37 (54.4 %)	
pTNM stage										
I, II	162	64 (39.5 %)	98 (60.5 %)	0.050	55 (34.0 %)	107 (66.0 %)	0.891	59 (37.1 %)	100 (62.9 %)	0.399
III, IV	205	102 (49.8 %)	103 (50.2 %)		71 (34.6 %)	134 (65.4 %)		85 (41.5 %)	120 (58.5 %)	
MSI status										
MSS/MSI-L	323	141 (43.7 %)	182 (56.3 %)	0.490	105 (32.5 %)	218 (67.5 %)	0.019	177 (54.8 %)	146 (45.2 %)	<0.001
MSI-H	32	16 (50.0 %)	16 (50.0 %)		17 (53.1 %)	15 (46.9 %)		28 (87.5 %)	4 (12.5 %)	
Chemotherapy status										
none	97	41 (42.3 %)	56 (57.7 %)	<0.001	67 (69.1 %)	30 (30.9 %)	0.748	49 (50.5 %)	48 (49.5 %)	0.175
pre-	0	0 (0.0 %)	0 (0.0 %)		0 (0.0 %)	0 (0.0 %)		0 (0.0 %)	0 (0.0 %)	
post-	269	177 (65.8 %)	92 (34.2 %)		181 (67.3 %)	88 (32.7 %)		162 (60.2 %)	107 (39.8 %)	
Pre- and post-	1	1 (100.0 %)	0 (0.0 %)		1 (100.0 %)	0 (0.0 %)		1 (100.0 %)	0 (0.0 %)	

*P*-values are calculated by using χ^2^-test or Fisher’s exact test

*Abbreviations*: *CRC* colorectal cancer, *T* tumor, *LG* low grade, *HG* high grade, *LN* lymph node, *MSS* microsatellite stable, *MSI-L* microsatellite instability-low, *MSI-H* microsatellite instability-high, *IHC* immunohistochemistry, *ISH* in-situ hybridization

### Correlation between c-MYC and ß-catenin expression in consecutive CRC patients

In cohort 1, c-MYC protein overexpression was correlated with mRNA overexpression (ρ, 0.479; *P* < 0.001), which was classified as moderate correlation [30]. ß-catenin nuclear expression was weakly associated with c-MYC protein overexpression and mRNA overexpression (ρ, 0.282; *P* < 0.001 and 0.211; *P* < 0.001, respectively).

### Locational heterogeneity of c-MYC and ß-catenin status

For analysis the locational heterogeneity of c-MYC and ß-catenin expression, we investigated cancer from three lesion, including the primary, distant and lymph node metastasis (cohort 2). All 176 cases had distant metastatic lesions. Among them, 142 cases had lymph node metastases, even though we dissected more than 20 lymph nodes in all CRC patients respectively. The clinicopathological features of the cohort 2 are indicated in Table 2 as previously reported [31]. Not every cohort 2 patients are stage IV due to metachronous metastasis which develops consequently after treatment of the first primary tumor. The distant metastatic sites were described below: liver in 82 cases (46.6 %), lung in 37 cases (21.0 %), peritoneal seeding in 38 cases (21.6 %), distant lymph nodes in 3 cases (1.7 %), and ovary in 16 cases (9.0 %).

**Table 2:** The clinicopathologic parameters of 176 advanced CRC patients with synchronous or metachronous metastases (cohort2)

		Total (*n* = 176)	Metastasis		
			Synchronous (*n* = 118)	Metachronous (*n* = 58)	*P*-Value
Sex	male	96	56 (47.5 %)	40 (69.0 %)	0.006
	female	80	62 (52.5 %)	18 (31.0 %)	
Metastatic site	liver	82	61 (52.1 %)	21 (36.2 %)	<0.001
	lung	37	10 (8.5 %)	27 (46.6 %)	
	peritoneal seeding	38	32 (26.5 %)	6 (10.3 %)	
	distant lymph node	3	2 (1.7 %)	1 (1.7 %)	
	ovary	16	13 (11.1 %)	3 (5.2 %)	
Location of primary tumor	appendix	1	1 (0.9 %)	0 (0.0 %)	0.293
	cecum	7	7 (6.0 %)	0 (0.0 %)	
	ascending colon	17	14 (12.0 %)	3 (5.2 %)	
	hepatic flexure	13	9 (7.7 %)	4 (6.9 %)	
	transverse colon	9	7 (6.0 %)	2 (3.4 %)	
	splenic flexure	7	4 (3.4 %)	3 (5.2 %)	
	descending colon	8	6 (5.1 %)	2 (3.4 %)	
	sigmoid colon	50	28 (23.9 %)	22 (37.9 %)	
	rectum	64	42 (35.0 %)	22 (37.9 %)	
T stage	T1	1	1 (0.9 %)	0 (0.0 %)	<0.001
	T2	3	1 (0.9 %)	2 (3.4 %)	
	T3	107	60 (50.4 %)	47 (81.0 %)	
	T4	65	56 (47.9 %)	9 (15.5 %)	
N stage	N0	34	13 (11.1 %)	21 (36.2 %)	<0.001
	N1	62	37 (31.6 %)	25 (43.1 %)	
	N2	80	68 (57.3 %)	12 (20.7 %)	
Stage	I	2	0 (0.0 %)	2 (3.4 %)	<0.001
	II	18	0 (0.0 %)	18 (31.0 %)	
	III	45	8 (6.8 %)	37 (63.8 %)	
	IV	111	110 (93.2 %)	1 (1.7 %)	
Differentiation	low grade	158	104 (88.0 %)	54 (93.1 %)	0.299
	high grade	18	14 (12.0 %)	4 (6.9 %)	
Lymphatic invasion	absent	62	38 (32.5 %)	24 (41.4 %)	0.247
	present	114	80 (67.5 %)	34 (58.6 %)	
Venous invasion	absent	123	77 (65.0 %)	46 (79.3 %)	0.052
	present	53	41 (35.0 %)	12 (20.7 %)	
Perineural invasion	absent	90	56 (47.9 %)	34 (58.6 %)	0.180
	present	86	62 (52.1 %)	24 (41.4 %)	
Tumor border	expanding	13	6 (5.1 %)	7 (12.1 %)	0.099
	infiltrative	163	112 (94.9 %)	51 (87.9 %)	

*P*-values are calculated by using χ^2^-test or Fisher’s exact test

*Abbreviations*: *T* tumor; *N* lymph node

In the primary tumors of cohort 2, c-MYC protein overexpression, mRNA overexpression and nuclear ß-catenin expression was detected in 57.6 % (102 out of 176), 77.4 % (137 out of 176) and 61.0 % (108 out of 176) of tumors, respectively. In distant metastatic tumors, c-MYC protein overexpression, mRNA overexpression, and nuclear ß-catenin expression was detected in 37.3 % (66 out of 176), 74.6 % (132 out of 176) and 47.5 % (84 out of 176) of tumors, respectively. In 142 lymph node metastases, we performed c-MYC and ß-catenin analysis in 111 cases which paraffin blocks were available. c-MYC protein overexpression, mRNA overexpression, and nuclear ß-catenin expression was detected in 66.7 % (74 out of 111), 77.5 % (86 out of 111) and 58.6 % (65 out of 111) of tumors, respectively.

The locational heterogeneity of c-MYC and ß-catenin status is demonstrated in Table 3. Discordance of c-MYC protein overexpression between the primary and distant metastatic cancer was detected in 45.5 % (80 out of 176) of cases, and discordance between the primary and lymph node metastatic cancer was observed in 31.5 % (35 out of 111) of cases. Discordance of *c-MYC* mRNA overexpression between the primary and distant metastatic cancer was detected in 25.6 % (45 out of 176) of cases, and discordance between the primary and lymph node metastatic cancer was observed in 30.6 % (34 out of 111) of cases. Discordance of nuclear ß-catenin expression between the primary and distant metastatic cancer was detected in 29.0 % (51 out of 176) of cases, while discordance between the primary and lymph node metastatic cancer was observed in 26.1 % (29 out of 111) of cases. Consequently, locational heterogeneity of c-MYC and ß-catenin expression was frequently seen in advanced CRC.

**Table 3:** Heterogeneity of c-MYC and ß-catenin with respect to tumor location in advanced CRC (cohort 2)

cMYC IHC		Distant metastasis		LN metastasis	
		Negative	Positive	Negative	Positive
Primary	Negative	52 (29.5 %)	22 (12.5 %)	23 (20.7 %)	21 (18.9 %)
	Positive	58 (33.0 %)	44 (25.0 %)	14 (12.6 %)	53 (47.7 %)
*cMYC* mRNA ISH		Distant metastasis		LN metastasis	
		Negative	Positive	Negative	Positive
Primary	Negative	19 (10.8 %)	20 (11.4 %)	8 (7.2 %)	17 (15.3 %)
	Positive	25 (14.2 %)	112 (63.6 %)	17 (15.3 %)	69 (62.2 %)
ß-catenin IHC		Distant metastasis		LN metastasis	
		Negative	Positive	Negative	Positive
Primary	Negative	55 (31.3 %)	14 (8.0 %)	30 (27.0 %)	13 (11.7 %)
	Positive	37 (21.0 %)	70 (39.8 %)	16 (14.4 %)	52 (46.8 %)

*Abbreviations*: *IHC* immunohistochemistry, *ISH* in-situ hybridization

### Prognostic impact of c-MYC and ß-catenin expression in CRC

**Fig. 2:**
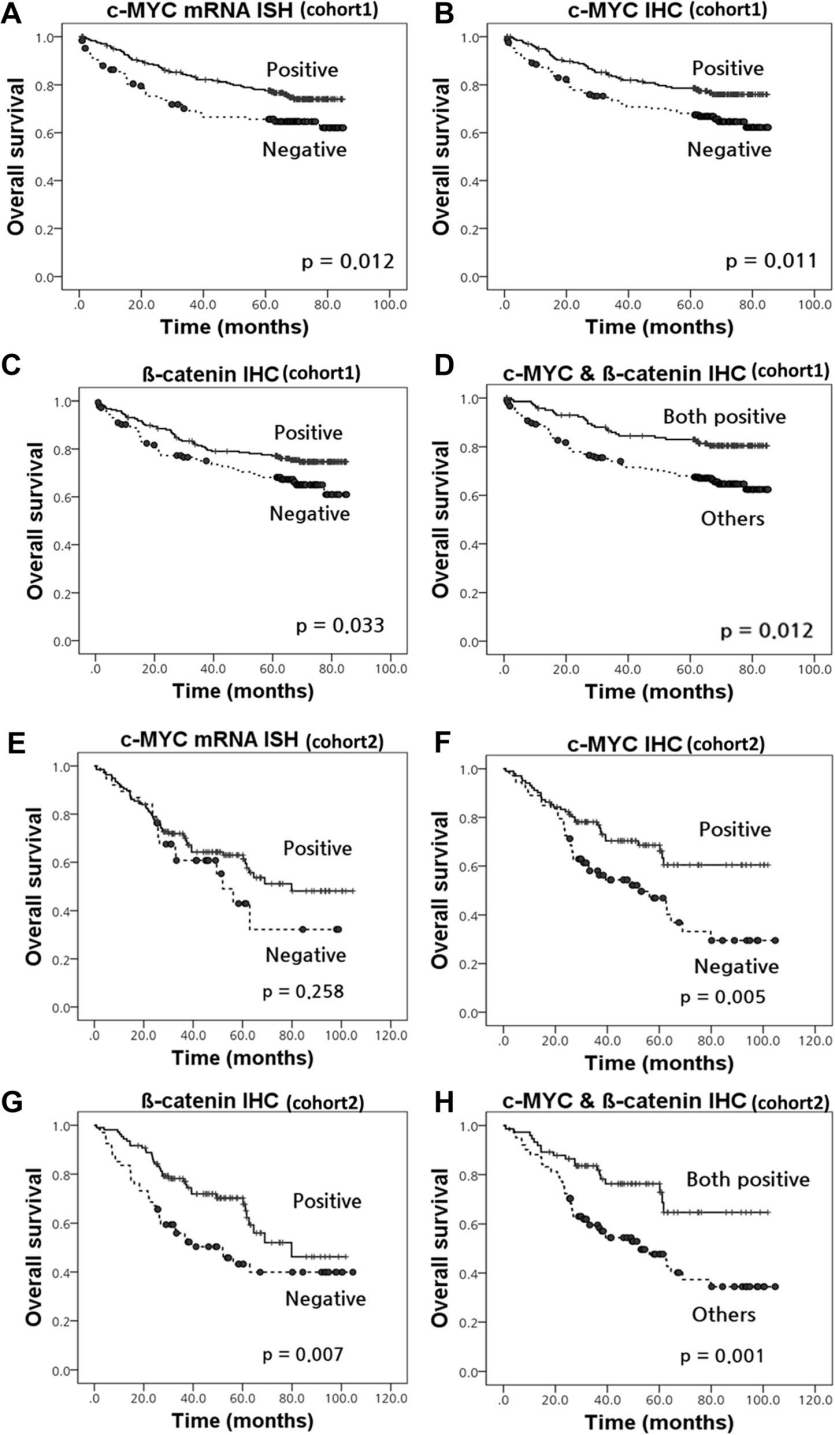
Kaplan–Meier survival curves illustrating the prognostic effects of c-MYC status in colorectal cancer. **a**-**d** Cohort 1; **a** *c-MYC* mRNA overexpression; **b** c-MYC protein overexpression; **c** Nuclear ß-catenin expression; **d** Co-expression of c-MYC and ß-catenin; **e**-**h** Primary tumor of cohort 2; **e** *c-MYC* mRNA overexpression; **f** c-MYC protein overexpression; **g** Nuclear ß-catenin expression; **h** Co-expression of c-MYC and ß-catenin

All CRC patients of our study were successfully included survival analysis (Fig. 2 and Additional file 1: Table S1). In the consecutive cohort (cohort 1), the median follow-up was 55 months (1–85 months) as previous reported [26]. c-MYC protein overexpression, mRNA overexpression and nuclear ß-catenin expression were significantly correlated with an improved survival in Kaplan-Meier analysis (*P* = 0.011, *P* = 0.012 and *P* = 0.033, respectively). When prognostic analysis was performed using the combined status of c-MYC and ß-catenin expression, positivity for both proteins (c-MYC/ß-catenin: +/+) was observed 84/367 (22.9 %) cases and was significantly correlated with an improved survival (*P* = 0.012). We additionally investigated the c-MYC protein overexpression by density of staining - scoring each tumor as low (0–1) to high (2–3) in cohort 1. The percentage of positive neoplastic cells was correlated with density of staining of neoplastic cells (ρ, 0.789; *P* < 0.001), which was categorized as strong correlation [30]. However, the staining density of c-MYC protein was not significantly correlated with patients’ prognosis (*P* = 0.070, Additional file 2: Figure S1).

In the cohort with metastases (cohort 2), the median follow-up was 43 months (1–105 months), as previous reported [31]. In the primary cancer, Kaplan-Meier analysis showed that c-MYC protein overexpression and nuclear ß-catenin expression were significantly correlated with improved prognosis (*P* = 0.005 and *P* = 0.007, respectively), but mRNA overexpression was not (*P* = 0.258). Co-expression of c-MYC and ß-catenin (c-MYC/ß-catenin: +/+) also predicted better prognosis (*P* = 0.001). The c-MYC and ß-catenin expression status in distant or lymph node metastatic cancer did not significantly associated with patients’ survival (*P* > 0.05, data not shown). The presence of locational heterogeneity of c-MYC and ß-catenin expression was not associated with survival (*P* > 0.05; data not shown). In addition, we evaluated the Kaplan–Meier survival for MSI status, stage, chemotherapy status, site of primary cancer and site of distant metastasis (Additional file 2: Figure S1).

The multivariate Cox’s proportional hazards regression model of c-MYC and ß-catenin expression was described in Table 4, and indicated that co-expression of c-MYC and ß-catenin was an independent prognostic factor for better survival in both cohort 1 and primary tumor of cohort 2 (*P* = 0.048 and *P* = 0.002, respectively). However, individual analysis of c-MYC protein overexpression, mRNA overexpression, and nuclear ß-catenin expression did not independently predict better prognosis.

**Table 4:** Multivariate Cox proportional hazard models for the predictors of overall survival

Factors	Univariate survival analysis		Multivariate survival analysis	
	HR (95 % CI)	*P* value	HR (95 % CI)	*P* value
Cohort 1				
co-expression of c-MYC and ß-catenin	0.482 (0.310–0.749)	0.012	0.629 (0.397–0.996)	0.048
Age	1.026 (1.008–1.045)	0.005	1.022 (1.004–1.040)	0.015
Size	1.244 (1.059–1.244)	0.001	1.049 (0.946–1.163)	NS (0.362)
Histologic grade (high vs. low)	3.143 (1.904–5.188)	<0.001	2.842 (1.608–5.023)	<0.001
Stage (3/4 vs. 1/2)	6.151 (3.494–10.829)	<0.001	2.942 (1.538–5.628)	0.001
Lymphatic invasion	3.661 (2.242–5.980)	<0.001	1.338 (0.763–2.349)	NS (0.310)
Perineural invasion	3.942 (2.648–5.870)	<0.001	2.337 (1.487–3.673)	<0.001
Venous invasion	3.985 (2.671–5.946)	<0.001	2.163 (1.390–3.366)	0.001
c-MYC mRNA expression	0.599 (0.403–0.891)	0.011	0.703 (0.464–1.066)	NS (0.097)
Age	1.026 (1.008–1.045)	0.005	1.024 (1.006–1.042)	0.008
Size	1.244 (1.059–1.244)	0.001	1.056 (0.953–1.169)	NS (0.297)
Histologic grade (high vs. low)	3.143 (1.904–5.188)	<0.001	2.785 (1.562–4.968)	0.001
Stage (3/4 vs. 1/2)	6.151 (3.494–10.829)	<0.001	3.017 (1.574–5.782)	0.001
Lymphatic invasion	3.661 (2.242–5.980)	<0.001	1.327 (0.756–2.330)	NS (0.324)
Perineural invasion	3.942 (2.648–5.870)	<0.001	2.438 (1.554–3.827)	<0.001
Venous invasion	3.985 (2.671–5.946)	<0.001	2.102 (1.356–3.259)	0.001
c-MYC protein expression	0.593 (0.400–0.880)	0.010	0.751 (0.498–1.132)	NS (0.171)
Age	1.026 (1.008–1.045)	0.005	1.023 (1.005–1.041)	0.012
Size	1.244 (1.059–1.244)	0.001	1.054 (0.951–1.169)	NS (0.317)
Histologic grade (high vs. low)	3.143 (1.904–5.188)	<0.001	2.967 (1.681–5.236)	<0.001
Stage (3/4 vs. 1/2)	6.151 (3.494–10.829)	<0.001	2.959 (1.549–5.653)	0.001
Lymphatic invasion	3.661 (2.242–5.980)	<0.001	1.344 (0.766–2.356)	NS (0.303)
Perineural invasion	3.942 (2.648–5.870)	<0.001	2.388 (1.522–3.746)	<0.001
Venous invasion	3.985 (2.671–5.946)	<0.001	2.105 (1.356–3.269)	0.001
ß-catenin protein expression	0.666 (0.449–0.986)	0.042	0.740 (0.497–1.101)	NS (0.138)
Age	1.026 (1.008–1.045)	0.005	1.023 (1.005–1.041)	0.012
Size	1.244 (1.059–1.244)	0.001	1.068 (0.966–1.180)	NS (0.197)
Histologic grade (high vs. low)	3.143 (1.904–5.188)	<0.001	2.897 (1.645–5.102)	<0.001
Stage (3/4 vs. 1/2)	6.151 (3.494–10.829)	<0.001	3.034 (1.587–5.801)	0.001
Lymphatic invasion	3.661 (2.242–5.980)	<0.001	1.356 (0.771–2.383)	NS (0.290)
Perineural invasion	3.942 (2.648–5.870)	<0.001	2.367 (1.508–3.717)	<0.001
Venous invasion	3.985 (2.671–5.946)	<0.001	2.071 (1.331–3.224)	0.001
Primary tumor of cohort 2				
co-expression of c-MYC and ß-catenin	0.430 (0.254–0.726)	0.001	0.440 (0.259–0.747)	0.002
Age	1.023 (1.001–1.045)	0.037	1.025 (1.004–1.047)	0.020
Stage (3/4 vs. 1/2)	7.894 (1.922–32.418)	0.004	5.731 (1.380–23.800)	0.016
Lymphatic invasion	2.480 (1.402–4.386)	0.002	1.712 (0.940–3.117)	NS (0.079)
Perineural invasion	2.119 (1.313–3.421)	0.002	1.724 (1.049–2.831)	0.032

*P*-values are calculated by using χ^2^-test or Fisher’s exact test

*Abbreviations*: *HR* hazard ratio

## Discussion

There were several studies on c-MYC status in various malignancies. Some malignant tumors with c-MYC overexpression including gastric carcinoma, esophageal squamous cell carcinoma, and soft tissue leiomyosarcoma are associated with poor survival [32–34]. Likewise, several cancers with *c-MYC* gene amplification tend to be correlated with poor survival [35–37]. Interestingly, *c-MYC* mRNA overexpression in CRC was reported to be correlated with improved survival [5], but this was opposite result to previous other study [38]. Nevertheless, recent research indicated that immunohistochemical c-MYC overexpression was significantly associated with better prognosis of CRC patients in univariate model, but not in multivariate model [19]. In addition, many studies have shown that ß-catenin is crucial part of the Wnt signaling pathway in CRC development [39]. Recently, a meta-analysis study showed that nuclear overexpression of ß-catenin appeared to be associated with progressive disease for CRC patients [20]. However, prognostic value of nuclear overexpression of ß-catenin in CRC patients remains controversial [40, 41].

In our study, overexpression of c-MYC protein in the consecutive cohort was significantly correlated with an improved prognosis in univariate model, but not in multivariate model. The prognostic significance of nuclear ß-catenin expression is similar to that of c-MYC protein overexpression. We performed a combined analysis of c-MYC and ß-catenin expression because these proteins are closely related. Astonishingly, co-expression of c-MYC and ß-catenin correlated with an improved prognosis by univariate and multivariate analysis. Although the advanced cohort was mainly consisted of stage IV CRC patients (111 cases; 63.1 %), co-expression of c-MYC and ß-catenin was independently predicted favorable prognosis. Furthermore, overexpression of c-MYC and ß-catenin—except c-MYC mRNA—in the advanced cohort was significantly correlated with better prognosis using a univariate model. Consequently, co-expression of c-MYC and ß-catenin determined by IHC might be of use in the assessment of CRC patients.

We also demonstrated that ß-catenin nuclear expression significantly associated with its target molecule c-MYC in CRC patients (ρ, 0.282; *P* < 0.001). Overexpression of c-MYC can be caused by complex regulatory pathways and multiple communications with other factors, rather than just *c-MYC* gene alterations [42]. An example of such a mechanism is signaling via ß-catenin, a c-MYC regulator whose nuclear accumulation is correlated with c-MYC overexpression [7, 43]. ß-catenin increases in the cytoplasm and undergoes translocation to the nucleus, where it plays as a transcription factor for target genes such as *c-MYC* [16]. These processes explain that nuclear expression of ß-catenin is partly responsible for c-MYC overexpression. As a result, co-expression of c-MYC and ß-catenin can be considered as c-MYC overexpression via ß-catenin in CRC. The *APC* gene mutation is the initial step of CRC oncogenesis [44] and often lead to deregulation of ß-catenin [45]. Thus, ß-catenin-dependent c-MYC overexpression can be suggested in early colorectal carcinogenesis. In addition, recent studies suggested that high-level nuclear β-catenin in CRC was significantly correlated with high Ki67 expression [46], and indicated that tumor proliferative activity was inversely related to CRC aggressiveness and metastases [47, 48]. For this reason, c-MYC overexpression via ß-catenin might have an influence on improved prognosis of CRC patients. Moreover, E Melo *et al*. reported that CpG island methylation interrupts several Wnt target genes, including *ASCL2* and *LGR5* during CRC progression and promoter methylation of Wnt target genes is a powerful predictive factor for CRC relapse [16]. Therefore, silencing of ß-catenin/Wnt pathway by methylation generates CRC progression and worse prognosis. It is noteworthy that our result adds clinical evidence to support these previous studies [16–18, 46–48]. The lack of ß-catenin expression in CRC patients with presenting c-MYC overexpression shows a rather worse survival, presumably because, in these patients, the c-MYC is controlled by other regulatory factors. Future studies will be required to dissect the different mechanisms of ß-catenin-mediated c-MYC overexpression.

In the advanced cohort, regional heterogeneity of c-MYC and ß-catenin expression was frequently observed in advanced CRC. Nonetheless, c-MYC and ß-catenin expression was associated with better prognosis in the primary, not distant and lymph node metastatic cancer. Consequently, when we evaluate prognosis with c-MYC and ß-catenin, tissue from primary CRC should be used. In daily practice for pathologists, metastatic cancer tissue can only occasionally be obtained. Some researchers suggest that regional heterogeneity should be considered as a potential limitation to the evaluation of prognostic and therapeutic value in tissue from metastatic lesions [49, 50]. Therefore, the importance of regional heterogeneity must be assessed in biomarker research.

## Conclusions

Our study comprehensively evaluated the c-MYC and ß-catenin status of CRC patients. Overexpression of c-MYC protein, mRNA, and ß-catenin nuclear expression were observed in 54.8, 65.7, and 60.2 % of consecutive CRC patients, respectively. c-MYC protein overexpression was significantly correlated with mRNA overexpression and with ß-catenin nuclear expression. Interestingly, co-expression of c-MYC and ß-catenin—in other words, c-MYC overexpression via ß-catenin—was an independent improved prognostic factor in both the consecutive and advanced cohort. These findings indicate that c-MYC and ß-catenin IHC can be used as prognostic marker of CRC patients. However, further investigations on the detailed mechanism of connection between c-MYC and ß-catenin and its impact on patients’ outcome in CRC are needed.

## Additional files

Additional file 1: Table S1. The number at risk in each category, at each interval for all Kaplan-Meier plots in Fig. 2. (DOCX 21 kb) Additional file 2: Figure S1. Kaplan–Meier survival curves illustrating the prognostic effects of clinicopathological parameter. (A-E) Cohort 1; (A) MSI status; (B) stage I, II versus III, IV; (C) chemotherapy status; (D) site of primary cancer; (E) c-MYC protein overexpression by staning density (F) Primary of tumor of cohort 2; site of distant metastasis. (TIF 416 kb) Additional file 3: Raw data of c-MYC and ß-catenin status obtained from cohort1. (XLSX 51 kb) Additional file 4: Raw data of c-MYC and ß-catenin status obtained from cohort2. (XLSX 21 kb)

## Abbreviations

CRC: Colorectal cancer
IHC: Immunohistochemistry
FFPE: Formalin-fixed and paraffin-embedded
MSI: Microsatellite instability
PCR: Polymerase chain reaction
HR: Hazard ratio
CI: Confidence interval
