# Supplementary material for: Favorable prognosis in colorectal cancer patients with co-expression of c-MYC and ß-catenin
Source: BMC Cancer. 2016 Sep 13;16(1):730. doi: 10.1186/s12885-016-2770-7 (PMC5020485; doi:10.1186/s12885-016-2770-7)
Supplement: Additional file 1: Table S1. — The number at risk in each category, at each interval for all Kaplan-Meier plots in Fig. 2. (DOCX 21 kb) [file 12885_2016_2770_MOESM1_ESM.docx]

Supplementary table 1. The number at risk in each category, at each interval for all Kaplan-

Meier plots in Figure 2.

|  | | Cohort1 | | | Cohort2 | | |
| --- | --- | --- | --- | --- | --- | --- | --- |
|  |  | Time period (months) | At risk | Died | Time period (months) | At risk | Died |
| c-MYC mRNA ISH | negative | 20 | 120 | 25 | 20 | 39 | 6 |
|  |  | 40 | 101 | 15 | 40 | 33 | 8 |
|  |  | 60 | 86 | 1 | 60 | 25 | 3 |
|  |  | 80 | 85 | 2 | 80 | 22 | 1 |
|  |  | 100 | 83 | 0 | 100 | 21 | 0 |
|  | positive | 20 | 241 | 24 | 20 | 137 | 22 |
|  |  | 40 | 217 | 18 | 40 | 115 | 24 |
|  |  | 60 | 199 | 9 | 60 | 91 | 1 |
|  |  | 80 | 190 | 6 | 80 | 90 | 7 |
|  |  | 100 | 184 | 0 | 100 | 84 | 0 |
| c-MYC IHC | negative | 20 | 166 | 29 | 20 | 74 | 12 |
|  |  | 40 | 137 | 18 | 40 | 62 | 20 |
|  |  | 60 | 119 | 5 | 60 | 42 | 3 |
|  |  | 80 | 114 | 4 | 80 | 39 | 5 |
|  |  | 100 | 110 | 0 | 100 | 34 | 0 |
|  | positive | 20 | 201 | 20 | 20 | 102 | 16 |
|  |  | 40 | 181 | 27 | 40 | 86 | 12 |
|  |  | 60 | 154 | 5 | 60 | 74 | 1 |
|  |  | 80 | 149 | 4 | 80 | 73 | 3 |
|  |  | 100 | 145 | 0 | 100 | 70 | 0 |
| ß-catenin IHC | negative | 20 | 146 | 26 | 20 | 68 | 18 |
|  |  | 40 | 120 | 11 | 40 | 50 | 14 |
|  |  | 60 | 109 | 7 | 60 | 36 | 3 |
|  |  | 80 | 102 | 4 | 80 | 33 | 1 |
|  |  | 100 | 98 | 0 | 100 | 32 | 0 |
|  | positive | 20 | 221 | 23 | 20 | 108 | 10 |
|  |  | 40 | 198 | 22 | 40 | 98 | 18 |
|  |  | 60 | 176 | 4 | 60 | 80 | 1 |
|  |  | 80 | 172 | 4 | 80 | 79 | 7 |
|  |  | 100 | 168 | 0 | 100 | 72 | 0 |
| c-MYC & ß-catenin IHC | others | 20 | 283 | 39 | 20 | 102 | 19 |
|  |  | 40 | 244 | 21 | 40 | 83 | 25 |
|  |  | 60 | 223 | 9 | 60 | 58 | 4 |
|  |  | 80 | 214 | 5 | 80 | 54 | 5 |
|  |  | 100 | 209 | 0 | 100 | 49 | 0 |
|  | both positive | 20 | 84 | 10 | 20 | 74 | 9 |
|  |  | 40 | 74 | 12 | 40 | 65 | 7 |
|  |  | 60 | 62 | 2 | 60 | 58 | 0 |
|  |  | 80 | 60 | 3 | 80 | 58 | 3 |
|  |  | 100 | 57 | 0 | 100 | 55 | 0 |
