# Supplementary figures and images for: Favorable prognosis in colorectal cancer patients with co-expression of c-MYC and ß-catenin
Source: BMC Cancer. 2016 Sep 13;16(1):730. doi: 10.1186/s12885-016-2770-7 (PMC5020485; doi:10.1186/s12885-016-2770-7)

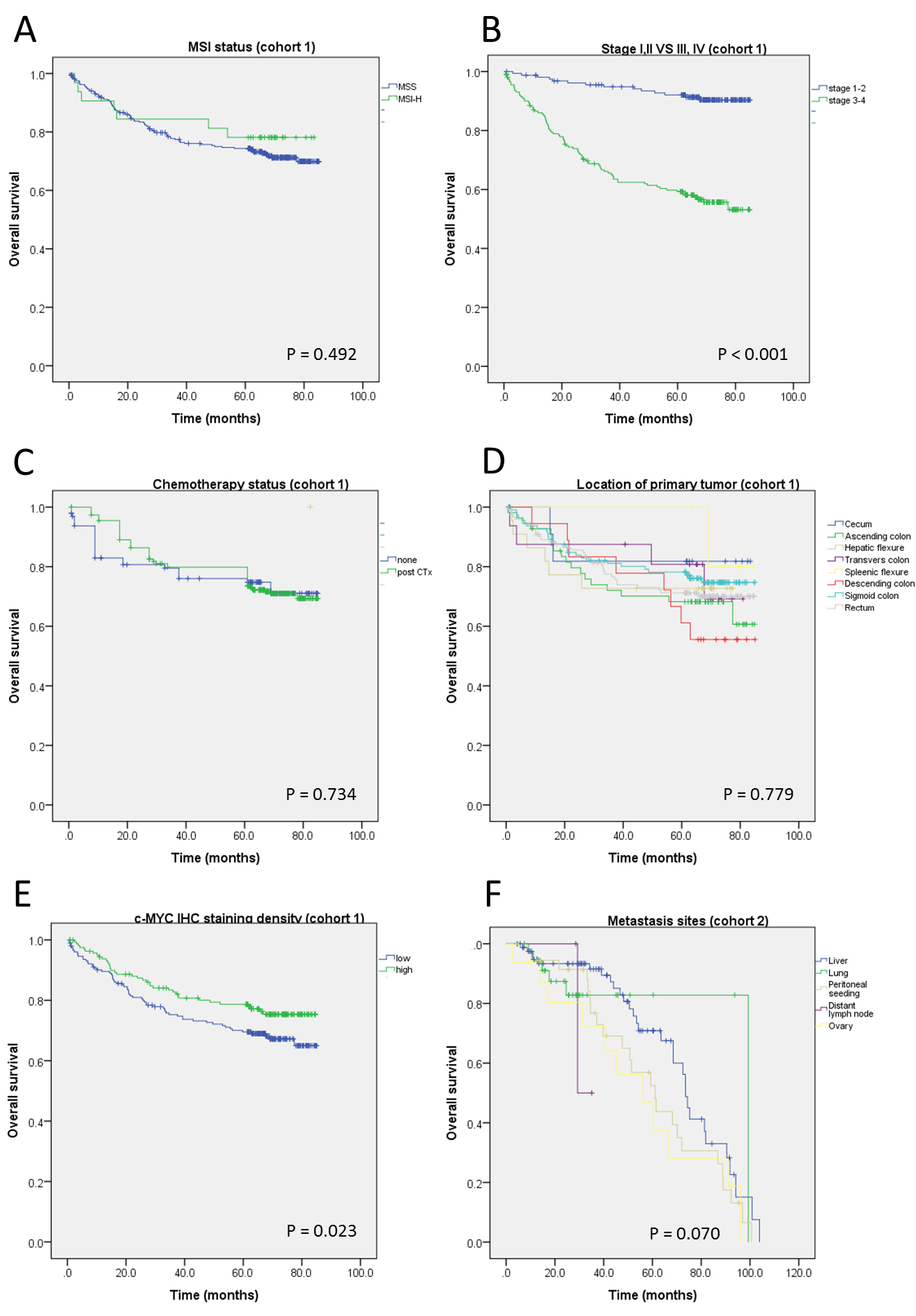

Supplement: Additional file 2: Figure S1. — Kaplan–Meier survival curves illustrating the prognostic effects of clinicopathological parameter. (A-E) Cohort 1; (A) MSI status; (B) stage I, II versus III, IV; (C) chemotherapy status; (D) site of primary cancer; (E) c-MYC protein overexpression by staning density (F) Primary of tumor of cohort 2; site of distant metastasis. (TIF 416 kb) [file 12885_2016_2770_MOESM2_ESM.tif]
